# Bacteriological Profile and Antibiotic Susceptibility in Patients With Diabetic Foot Infections

**DOI:** 10.7759/cureus.82809

**Published:** 2025-04-22

**Authors:** Eligio Copari-Vargas, Luisa Elena Copari-Vargas, Tania Libertad Copari-Vargas, Luis Fernando Domínguez-Valdez, Eligio Copari-Jimenez, Juan Guillermo Urquizo-Ayala

**Affiliations:** 1 Endocrinology, Diabetes and Metabolism, Hospital General de México "Dr. Eduardo Liceaga", Mexico City, MEX; 2 Internal Medicine, Hospital de Clínicas, La Paz, BOL; 3 Internal Medicine, Unidad Médica de Alta Especialidad (UMAE) Hospital de Especialidades “Dr. Antonio Fraga Mouret” Centro Médico Nacional “La Raza”, Mexico City, MEX

**Keywords:** antimicrobial resistance, diabetes, diabetic foot infection, diabetic foot ulcer, empiric antibiotic therapy

## Abstract

Introduction

Diabetic foot ulcer (DFU), is a common complication of uncontrolled diabetes, that frequently progresses to diabetic foot infection (DFI), contributing to morbidity, economic burden, and lower limb amputations. This study characterizes the bacteriological profile and antibiotic susceptibility patterns in DFIs patients.

Methods

A retrospective study was conducted from January 2014 and December 2018. All patients records with microbiologically confirmed DFIs. Demographic data, clinical history, ulcer classification, and microbiological findings were reviewed. Bacterial isolates were identified, and antibiotic susceptibility testing was performed.

Results

Of a total of 56 patients diagnosed with DFIs at a secondary care hospital in Bolivia, the medical records of 42 individuals were reviewed, of which 28 patients met the inclusion criteria for analysis. Among these, 89.2% (n=25) had polymicrobial infections. *Staphylococcus aureus* was the most frequently isolated pathogen, representing 38.4% (n=10) of cases, followed by *Enterococcus faecalis* and *Escherichia coli*. Among Gram-negative isolates, *Acinetobacter baumannii* exhibited complete resistance to both ampicillin and cephalosporins, highlighting its multidrug-resistant (MDR) profile. The most frequently administered antibiotics were metronidazole, ceftriaxone, and ciprofloxacin. Resistance to quinolones and β-lactam antibiotics was particularly pronounced across several isolates.

Conclusions

DFUs with associated infections (DFIs) in this study were predominantly polymicrobial and showed a high prevalence of MDR pathogens. These findings underscore the importance of early and accurate microbiological assessment to prevent infection progression and optimize antibiotic selection in patients with DFUs. Targeted antimicrobial therapy is essential to reduce the risk of treatment failure and amputation. Continued surveillance of resistance patterns is critical to inform empirical treatment strategies in similar healthcare settings.

## Introduction

Diabetes mellitus (DM) is a global health challenge affecting approximately 463 million adults worldwide, with projections reaching 700 million by 2045, positioning it among the most prevalent chronic diseases globally [1,2]. In Latin America, DM represents a major public health burden, with prevalence rates ranging from 8% to 13%, and reaching 7.2% in Bolivia [3,4]. Chronic hyperglycemia triggers oxidative stress and endothelial dysfunction, promoting both microvascular and macrovascular complications that predispose patients to diabetic foot ulcers (DFUs). These ulcers affect an estimated 9.1 to 26.1 million diabetic individuals annually and remain a leading cause of non-traumatic lower limb amputations [5]. Progression from DFU to diabetic foot infection (DFI) is a critical clinical concern, with up to 50-60% of ulcers becoming infected and approximately 80% of non-traumatic amputations attributable to DFIs [6]. Despite prolonged and repeated antibiotic therapies, many cases fail to resolve clinically, increasing the risk of infections with multidrug-resistant (MDR) pathogens, extended hospitalization, and treatment failure [7]. The pathogenesis of DFIs is multifactorial, involving peripheral neuropathy, ischemia, and immune dysregulation, which together create an environment highly susceptible to bacterial colonization and chronic infection [8]. Hyperglycemia-induced endothelial dysfunction and nitric oxide suppression contribute to delayed wound healing. Additionally, immune cells such as neutrophils and macrophages exhibit impaired chemotaxis, phagocytosis, and phenotypic switching, maintaining a chronic pro-inflammatory state. The accumulation of advanced glycation end-products (AGEs) and reactive oxygen species (ROS) further exacerbates tissue damage and inhibits regenerative cellular processes [9]. This study aims to characterize the microbiological profile and antimicrobial resistance patterns of DFIs in a secondary care hospital setting in Bolivia, with the goal of providing evidence-based guidance for antibiotic selection and clinical decision-making.

## Materials and methods

Between January 2014 and December 2018, 56 patients diagnosed with DFUs were identified at the Internal Medicine Units I and II of Hospital de Clínicas, La Paz, Bolivia. Inclusion criteria consisted of adult patients (>18 years) of both genders with a confirmed diagnosis of diabetes mellitus, the presence of a DFU, and microbiological culture and antibiogram results documented in their clinical history. Exclusion criteria included prior antibiotic use, incomplete hospitalization, immunosuppressive treatment, and missing data affecting study variables. The study protocol was reviewed and approved by the Committee for the Evaluation of Research Protocols for Medical Residents, Universidad Mayor de San Andrés, La Paz, with registration number BOL/LP-2482-2024.

Out of the 56 total patients, the files of 14 were not found, leaving 42 clinical records available. Of these, 14 were excluded; four did not meet the inclusion criteria, three had a different diagnosis confirmed after admission, five had missing data, and two did not complete hospitalization. The final study group consisted of 28 patient records. We analyzed the epidemiological characteristics and clinical history of patients, including age, sex, duration of diabetes mellitus, associated conditions, glycated hemoglobin levels, albuminuria, microalbuminuria, and the severity of DFUs using the Wagner Classification System [9]. Ulcers were categorized from Grade 0, indicating high risk without ulceration, to Grade 5, representing extensive gangrene. Microbiological findings were examined based on positive bacterial culture results from ulcer samples, identifying pathogen prevalence and distribution. Antibiotic susceptibility was determined through antibiogram testing, classifying bacterial responses as sensitive, resistant, or intermediate according to the efficacy of tested antibiotics. During hospitalization, the progression of DFUs and response to antibiotic treatment were evaluated.

The IBM SPSS Statistics for Windows, Version 25.0 (Released 2017; IBM Corp., Armonk, New York, United States) was used for data analysis. Descriptive statistics were applied to summarize the findings. Categorical variables, including sex and ulcer classification, were analyzed using frequencies and percentages. Quantitative variables, such as age and glycated hemoglobin levels, were described using mean and standard deviation or median with interquartile ranges, depending on the distribution of the data.

## Results

The mean age of the 28 study participants was 59.4 ± 2.3 years, with a gender distribution of 64.3% (n=18) female patients and 35.7% (n=10) male patients. Regarding the duration of type 2 diabetes mellitus (T2DM), 57.1% (n=16) had lived with the disease for 15 years. Diabetes-related complications were prevalent, with nephropathy affecting 85.7% (n=24), peripheral neuropathy 78.6% (n=22) and atherosclerosis 50% (n=14). The mean glycated hemoglobin (HbA1c) level was 8.7 ± 1.1%, indicating poor glycemic control. Albuminuria was observed in 21.4% (n=6) and microalbuminuria in 35.7% (n=10). DFUs were graded according to the Wagner classification system, with the most common presentations being Grade 2 and Grade 3, each accounting for 28.6% (n=8) of the cases. Sepsis at the time of admission was documented in 7.1% (n=2) of patients.

Regarding lifestyle-related risk factors, 28.6% (n=8) of patients reported active tobacco use, and 50% (n=14) consumed alcohol regularly (Table 1).

**Table 1:** Demographic and clinical characteristics of patients with diabetic foot T2DM: type 2 diabetes mellitus, HbA1c: glycated hemoglobin, n/a: not applicable

Character	Total patients (n=28)
Age, year (mean ± SD)	59.4 + 2.3
Sex, n (%)	n/a
Female	18 (64.3%)
Male	10 (35.7%)
Duration of TDM2, years, n (%)	n/a
5	6 (21.4%)
10	4 (14.3%)
15	16 (57.1%)
20	2 (7.1%)
Associated conditions, n (%)	n/a
Nephropathy	24 (85.7%)
Peripheral neuropathy	22 (78.6%)
Retinopathy	12 (42.9%)
Atherosclerosis	14 (50.0%)
Hypertension	4 (14.3%)
Dyslipidemia	22 (78.6%)
HbA1c (mean ± SD)	8.7 + 1.1
Albuminuria, n (%)	6 (21.4%)
Microalbuminuria, n (%)	10 (35.7%)
Diabetic Foot Classification (Wagner Scale), n (%)	n/a
Grade 0	4 (14.3%)
Grade 1	4 (14.3%)
Grade 2	8 (28.6%)
Grade 3	8 (28.6%)
Grade 4	2 (7.1%)
Grade 5	2 (7.1%)

Microbiological profile and antibiotic management in diabetic foot infections

Among the 28 ulcer cultures analyzed, 89.2% (n=25) demonstrated polymicrobial growth, while 10.7% (n=3) were monomicrobial isolates. Gram-positive bacteria were predominant, with *Staphylococcus aureus* identified as the most frequently isolated organism in 38.4% (n=10) of samples, followed by *Enterococcus faecalis* in 15.3% (n=4) and *Streptococcus agalactiae* in 7.6% (n=2). Regarding Gram-negative organisms, *Escherichia coli* 15.3% (n=4) was the most common isolate, followed by *Acinetobacter baumannii*, *Klebsiella aerogenes*, and *Proteus mirabilis*, each isolated in 7.6% (n=2) of cultures (Table 2).

**Table 2:** Characteristics of cultures found on patients with diabetic foot *S. aureus*: *Staphylococcus aureus*, *S. agalactiae*: *Streptococcus agalactiae*, *E. faecalis*: *Enterococcus faecalis*, *E. coli*: *Escherichia coli*, *A. baumannii*: *Acinetobacter baumannii*, *K. aerogenes*: *Klebsiella aerogenes*, *P. mirabilis*: *Proteus mirabilis*, n/a: not applicable

Culture characteristics	Total no. (%) (N = 28)
Monomicrobial, n (%)	3 (10.7%)
Polymicrobial, n (%)	25 (89.2%)
Gram-positive	n/a
S. aureus	10 (38.4%)
S. agalactiae	2 (7.6%)
E. faecalis	4 (15.3%)
Gram-negative	n/a
E. coli	4 (15.3%)
A. baumannii	2 (7.6%)
K. aerogenes	2 (7.6%)
P. mirabilis	2 (7.6%)

Empirical antibiotic therapy was initiated in all patients upon hospitalization. The most frequently administered antibiotics were metronidazole 18.7% (n=6), ceftriaxone 12.5% (n=4), ciprofloxacin 12.5% (n=4), and vancomycin 12.5% (n=4). Additional agents included amoxicillin-clavulanic acid 6.2% (n=2), cefotaxime 6.2% (n=2), imipenem 6.2% (n=2), gentamicin 6.2% (n=2), and doxycycline 6.2% (n=2). At discharge, the most commonly prescribed antibiotics were amoxicillin-clavulanic acid 12.5% (n=4), ciprofloxacin 10.7% (n=3), and doxycycline 6.2% (n=2), reflecting targeted adjustments based on microbiological findings (Table 3).

**Table 3:** Treatment used at admission and discharge for the management of diabetic foot ulcers n/a: not applicable

Treatment	Total no. (%) (N = 28)
Multitherapy antibiotic treatment, n (%)	3 (10.7%)
Monotherapy antibiotic treatment, n (%)	25 (89.2%)
Admission	n/a
Amikacin	2 (6.3%)
Amoxicillin-clavulanic acid	2 (6.2%)
Cefotaxime	2 (6.2%)
Ceftriaxone	4 (12.5%)
Ciprofloxacin	4 (12.5%)
Cloxacillin	2 (6.2%)
Doxycycline	2 (6.2%)
Gentamicin	2 (6.2%)
Imipenem	2 (6.2%)
Metronidazole	6 (18.7%)
Vancomycin	4 (12.5%)
Discharge	n/a
Amoxicillin-clavulanic acid	4 (12.5%)
Ciprofloxacin	3 (10.7%)
Doxycycline	2 (6.2%)

Antibiotic susceptibility and resistance

The antibiotic susceptibility analysis revealed high variability in resistance patterns among Gram-positive isolates, underscoring the microbiological complexity of DFIs and the critical need for pathogen-directed antimicrobial therapy. *Staphylococcus aureus* exhibited 100% susceptibility to clindamycin, gentamicin, and amikacin, while 80% remained susceptible to quinolones. However, resistance to cephalosporins was observed in 40%, and 20% of isolates were resistant to oxacillin, indicating a concerning trend toward β-lactam resistance. *Streptococcus agalactiae* demonstrated 100% susceptibility to ampicillin, clindamycin, and oxacillin, but 50% resistance to tetracycline, limiting its use. *Enterococcus faecalis* showed complete susceptibility to vancomycin and ampicillin, supporting their role as first-line treatments. Nonetheless, 50% of isolates exhibited resistance to clarithromycin, which may compromise the efficacy of macrolide-based regimens (Table 4).

**Table 4:** Antibiotic susceptibility and resistance of the Gram-positive microorganisms found in ulcer cultures of patients with diabetic foot S: Susceptible, R: Resistant, *S. aureus*: *Staphylococcus aureus*, *S. agalactiae*: *Streptococcus agalactiae*, *E. faecalis*: *Enterococcus faecalis*

Antibiotics	S. aureus		S. agalactiae		E. faecalis	
	S (%)	R (%)	S (%)	R (%)	S (%)	R (%)
Amikacin	2	n/a	n/a	n/a	n/a	n/a
Amoxicillin-clavulanic acid	n/a	n/a	n/a-	n/a	n/a	n/a
Ampicillin	n/a	n/a	2	n/a	4	n/a
Aztreonam	n/a	n/a	n/a	n/a	n/a	n/a
Azithromycin	n/a	n/a	n/a	n/a	n/a	2
Cephalosporins	3	4	n/a	n/a	n/a	n/a
Clarithromycin	n/a	n/a	n/a	n/a	n/a	2
Clindamycin	10	n/a	2	n/a	n/a	n/a
Chloramphenicol	2	n/a	n/a	n/a	n/a	n/a
Cotrimoxazole	3	2	n/a	n/a	n/a	n/a
Doxycycline	4	n/a	n/a	n/a	2	n/a
Gentamicin	5	n/a	n/a	n/a	n/a	4
Imipenem	n/a	n/a	n/a	n/a	n/a	n/a
Meropenem	n/a	n/a	n/a	n/a	n/a	n/a
Minocycline	2	n/a	n/a	n/a	n/a	n/a
Oxacillin	5	4	2	n/a	n/a	n/a
Quinolones	10	2	2	n/a	n/a	4
Tetracycline	n/a	2	n/a	n/a	n/a	n/a
Vancomycin	n/a	n/a	n/a	n/a	4	n/a

Among Gram-negative isolates, *Escherichia coli* exhibited 100% susceptibility to cephalosporins and gentamicin, while presenting 50% resistance to quinolones. *Acinetobacter baumannii* displayed 100% resistance to ampicillin and cephalosporins, confirming its multidrug-resistant (MDR) profile, which may require carbapenem or colistin-based treatment in severe cases. *Klebsiella aerogenes* and *Proteus mirabilis* exhibited 100% resistance to ampicillin, consistent with their intrinsic β-lactam resistance, while 50% of isolates remained susceptible to quinolones and cephalosporins, supporting their potential role in step-down therapy. The presence of multidrug-resistant *Acinetobacter baumannii* poses a significant clinical challenge, necessitating alternative therapies such as carbapenems or polymyxins for severe cases. Targeted therapy based on susceptibility profiles remains crucial in optimizing treatment outcomes in DFIs (Table 5).

**Table 5:** Antibiotic susceptibility and resistance of the gram-negative microorganisms found in ulcer cultures of patients with diabetic foot S: Susceptible, R: Resistant, *E. coli*: *Escherichia coli*, *A. baumannii*: *Acinetobacter baumannii*, *K. aerogenes*: *Klebsiella aerogenes*, *P. mirabilis*: *Proteus mirabilis*

Antibiotics	E. coli		A. baumannii		K. aerogenes		P. mirabilis	
	S (%)	R (%)	S (%)	R (%)	S (%)	R (%)	S (%)	R (%)
Amikacin	2	n/a	n/a	n/a	2	n/a	2	n/a
Amoxicillin-clavulanic acid	n/a	n/a	n/a	n/a	n/a	2	2	n/a
Ampicillin	n/a	2	n/a	2	n/a	n/a	n/a	n/a
Aztreonam	n/a	n/a	n/a	n/a	n/a	n/a	n/a	2
Azithromycin	n/a	n/a	n/a	n/a	n/a	n/a	n/a	n/a
Cephalosporins	4	n/a	n/a	2	2	2	n/a	2
Clarithromycin	n/a	n/a	n/a	n/a	n/a	n/a	n/a	n/a
Clindamycin	n/a	n/a	n/a	n/a	n/a	n/a	n/a	n/a
Chloramphenicol	n/a	n/a	n/a	n/a	n/a	n/a	n/a	n/a
Cotrimoxazole	2	n/a	n/a	n/a	n/a	n/a	n/a	2
Doxycycline	2	n/a	n/a	n/a	n/a	n/a	n/a	n/a
Gentamicin	4	n/a	n/a	n/a	n/a	n/a	n/a	n/a
Imipenem	n/a	n/a	n/a	2	n/a	n/a	n/a	n/a
Meropenem	n/a	n/a	n/a	2	n/a	n/a	n/a	n/a
Minocycline	n/a	n/a	n/a	n/a	n/a	n/a	n/a	n/a
Oxacillin	4	n/a	n/a	n/a	2	n/a	n/a	2
Quinolones	n/a	n/a	n/a	2	2	n/a	n/a	2
Tetracycline	n/a	n/a	n/a	n/a	n/a	n/a	n/a	n/a
Vancomycin	n/a	n/a	n/a	n/a	n/a	n/a	n/a	n/a

## Discussion

DFIs are a major cause of morbidity in individuals with diabetes, particularly among older adults [10]. These infections are often associated with DFUs, which frequently harbor complex polymicrobial infections comprising both aerobic and anaerobic organisms. Notably, pathogens such as *Staphylococcus aureus*, *Pseudomonas aeruginosa*, and *Streptococcus* species often coexist within biofilms that promote microbial synergy, increase virulence, and confer resistance to standard antibiotic regimens.

In addition to the microbiological burden, the host immune response in DFUs is severely impaired. Chronic hyperglycemia disrupts neutrophil chemotaxis, phagocytosis, and microbial killing. Macrophages exhibit a sustained pro-inflammatory (M1) phenotype, preventing the resolution of inflammation and impeding the transition to the reparative (M2) phase necessary for tissue regeneration [8]. These immune alterations establish a persistent inflammatory environment that delays wound healing and favors chronic infection. Other key factors that exacerbate DFIs include peripheral artery disease, foot deformities, diabetic neuropathy, unnoticed trauma, and increased plantar pressure. Moreover, chronic renal insufficiency and recurrent ulceration further predispose patients to severe infections [9].

In our study, the mean age of patients with DFIs was 59.4 years, aligning with previous research indicating a higher prevalence among older individuals with poor glycemic control. Elevated HbA1c, fasting blood glucose, or random blood glucose levels have been associated with an increased risk of ulceration and subsequent amputation [8]. A notable predominance of females, accounting for 64.3% of cases, contrasts with global trends that report a higher incidence among males [11]. This discrepancy may reflect biological, socioeconomic, and cultural factors specific to the Bolivian population. Most patients exhibited diabetes-related complications, including nephropathy in 85.7%, peripheral neuropathy in 78.6%, and atherosclerosis in 50%, all of which significantly increase the risk of severe DFIs [7].

This study revealed a high prevalence of polymicrobial infections, affecting 89.2% of cases. *Staphylococcus aureus* was the most frequently isolated pathogen at 38.4%, followed by *Enterococcus faecalis* and *Escherichia coli*, each accounting for 15.3%. These findings align with previous studies identifying *Staphylococcus aureus* as the primary etiological agent in DFIs [12]. Reported prevalence varies geographically, with higher rates in Mexico at 42% and Australia at 71.8%, but lower in Turkey at 11.4% [13]. The presence of both *Enterococcus faecalis* and *Escherichia coli* highlights the coexistence of Gram-positive and Gram-negative bacteria, reinforcing the polymicrobial nature of DFIs, particularly in developing regions [14].

Microbial interactions in polymicrobial infections facilitate the production of virulence factors, triggering inflammation, impairing wound healing, and contributing to infection chronicity [12, 15]. The skin’s native microbiota also plays a critical role. Commensals such as *Staphylococcus epidermidis* and *Corynebacterium* spp., typically non-pathogenic, may transition to opportunistic pathogens under diabetic immunocompromise, emphasizing the need to distinguish colonizers from true pathogens [16].

The detection of MDR Gram-negative organisms, particularly *Acinetobacter baumannii*, which showed complete resistance to ampicillin and cephalosporins, is a particularly worrisome trend. These findings mirror global reports of rising antimicrobial resistance in diabetic foot settings, which complicates therapeutic decision-making and worsens clinical outcomes [11]. Antimicrobial susceptibility analysis revealed that *Staphylococcus aureus* exhibited complete susceptibility to clindamycin, gentamicin, and amikacin, while showing 20% resistance to β-lactams, a critical factor for empirical therapy selection. Gram-negative pathogens demonstrated significant susceptibility to ceftazidime and gentamicin but showed notable resistance to quinolones, consistent with reports of increasing resistance to these agents. The presence of MDR pathogens, particularly *Acinetobacter baumannii*, underscores the need for targeted therapeutic strategies based on specific susceptibility profiles to improve clinical outcomes and reduce amputation rates. The polymicrobial nature of DFIs increases the risk of treatment failure, further highlighting the importance of individualized antimicrobial management [17].

Accurate identification of pathogenic microbes and differentiation from colonizing bacteria are essential for selecting appropriate antibiotic therapy. Empirical treatment for DFIs in patients at risk of polymicrobial infections includes ampicillin/sulbactam, ceftriaxone combined with either clindamycin or metronidazole, levofloxacin with clindamycin, moxifloxacin, and ertapenem [18]. Once culture and sensitivity data become available, therapy should be promptly adjusted to improve outcomes and mitigate resistance development.

This study has several limitations. As a single-center retrospective analysis, the findings may not be generalizable to all healthcare settings. Additionally, variability in microbiological techniques and susceptibility testing methods may affect the precision of pathogen identification. Nevertheless, our data provide valuable insights into the local epidemiology of DFUs, DFIs, and antibiotic resistance patterns, offering essential guidance for clinicians in optimizing empirical therapy and managing diabetic foot complications more effectively.

## Conclusions

DFUs with associated infections (DFIs) in this study were predominantly polymicrobial, with a high prevalence of multidrug-resistant pathogens, particularly *Acinetobacter baumannii*. The variability in antimicrobial susceptibility patterns highlights the need for routine microbiological surveillance and locally adapted empirical treatment. Establishing targeted antibiotic protocols based on regional resistance data is essential to improve clinical outcomes and limit the spread of antimicrobial resistance.
